# Complete genome sequence of *Chitinophaga pinensis* type strain (UQM 2034^T^)

**DOI:** 10.4056/sigs.661199

**Published:** 2010-02-28

**Authors:** Tijana Glavina Del Rio, Birte Abt, Stefan Spring, Alla Lapidus, Matt Nolan, Hope Tice, Alex Copeland, Jan-Fang Cheng, Feng Chen, David Bruce, Lynne Goodwin, Sam Pitluck, Natalia Ivanova, Konstantinos Mavromatis, Natalia Mikhailova, Amrita Pati, Amy Chen, Krishna Palaniappan, Miriam Land, Loren Hauser, Yun-Juan Chang, Cynthia D. Jeffries, Patrick Chain, Elizabeth Saunders, John C. Detter, Thomas Brettin, Manfred Rohde, Markus Göker, Jim Bristow, Jonathan A. Eisen, Victor Markowitz, Philip Hugenholtz, Nikos C. Kyrpides, Hans-Peter Klenk, Susan Lucas

**Affiliations:** 1 DOE Joint Genome Institute, Walnut Creek, California, USA; 2 DSMZ - German Collection of Microorganisms and Cell Cultures GmbH, Braunschweig, Germany; 3 Los Alamos National Laboratory, Bioscience Division, Los Alamos, New Mexico, USA; 4 Biological Data Management and Technology Center, Lawrence Berkeley National Laboratory, Berkeley, California, USA; 5 Oak Ridge National Laboratory, Oak Ridge, Tennessee, USA; 6 Lawrence Livermore National Laboratory, Livermore, California, USA; 7 HZI – Helmholtz Centre for Infection Research, Braunschweig, Germany; 8 University of California Davis Genome Center, Davis, California, USA

**Keywords:** filamentous, gliding, myxospores, aerobic, mesophile, Gram-negative, biomass degrader, chitinolytic, ‘*Chitinophagaceae’*, GEBA

## Abstract

*Chitinophaga pinensis* Sangkhobol and Skerman 1981 is the type strain of the species which is the type species of the rapidly growing genus *Chitinophaga* in the sphingobacterial family ‘*Chitinophagaceae*’. Members of the genus *Chitinophaga* vary in shape between filaments and spherical bodies without the production of a fruiting body, produce myxospores, and are of special interest for their ability to degrade chitin. Here we describe the features of this organism, together with the complete genome sequence, and annotation. This is the first complete genome sequence of a member of the family ‘*Chitinophagaceae*’, and the 9,127,347 bp long single replicon genome with its 7,397 protein-coding and 95 RNA genes is part of the *** G****enomic* *** E****ncyclopedia of* *** B****acteria and* *** A****rchaea * project.

## Introduction

Strain UQM 2034^T^ (DSM 2588 = ATCC 43595 = KCTC 3412) is the type strain of the species *Chitinophaga pinensis* and was first described in 1981 by Sangkhobol and Skerman [1]. In 1981, strain UQM 2034^T^ was described as a long, filamentous, gliding microorganism isolated from an infusion of litter from the base of a pine tree in Alderley, Brisbane, Australia [1]. In 1999, the phylogenetic position of *C. pinensis* was determined. The comparison of 16S rRNA sequences revealed *Flexibacter filimoris* as the most closely related bacterium [2].

In 2006 Kämpfer *et al*. reclassified *F. sancti*, *F. filiformis*, *F. japonensis* and *Cytophaga arvensicola* to the monospecific genus *Chitinophaga* and proposed *C. skermanii* sp. nov. [3]. In recent years the number of newly described species belonging to the genus *Chitinophaga* increased. Two additional new *Chitinophaga* species were described in 2007, *C. ginsengisegetis* sp. nov. and *C. ginsengisoli* sp. nov. isolated from soil of a ginseng field in South Korea [4]. In the same year Kim and Jung described the new species *C. terrae* sp. nov [5]. In 2009, three additional *Chitinophaga* species were described: *C. niabensis* sp. nov. [6], *C. niastensis* sp. nov [6], and *C. rupis* sp. nov [7]. Here we present a summary classification and a set of features for *C. pinensis* UQM 2034^T^, together with the description of the complete genomic sequencing and annotation.

## Classification and features

The most similar 16S rRNA gene sequences from cultivated strains that are stored in GenBank originate from isolates belonging to different species of the genus *Chitinophaga*: *C. sancti*, *C. filiformis* and *C. ginsengisoli* with 96-97% sequence similarity; all of them were isolated from soil samples. In metagenomic surveys of environmental samples only 16S rRNA genes with sequence similarity values below 92% to *C. pinensis* were detected, indicating that members of this species are not abundant in the so far genomically screened habitats (status July 2009).

**Figure 1:**
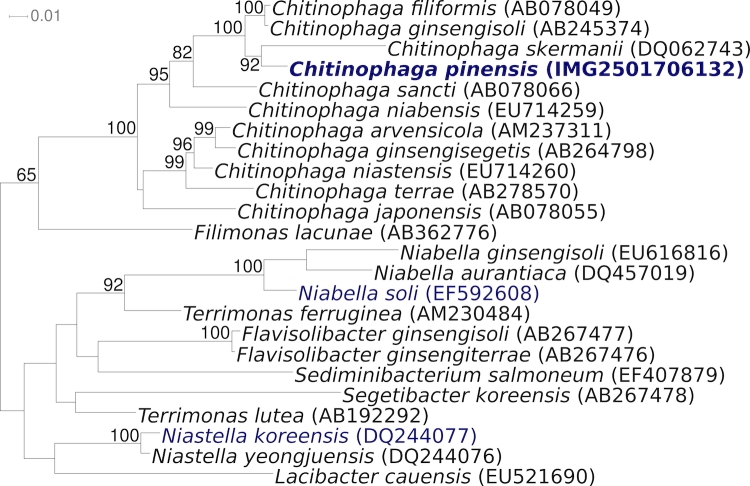
Phylogenetic tree highlighting the position of *C. pinensis* UQM 2034^T^ relative to the other type strains within the genus and selected other type strains within the family ‘*Chitinophagaceae*’. The tree was inferred from 1,410 aligned characters [8,9] of the 16S rRNA gene sequence under the maximum likelihood criterion [10] and rooted in accordance with the current taxonomy. The branches are scaled in terms of the expected number of substitutions per site. Numbers above branches are support values from 1,000 bootstrap replicates if larger than 60%. Lineages with type strain genome sequencing projects registered in GOLD [11] are shown in blue, published genomes in bold.

Figure 1 shows the phylogenetic neighborhood of *C. pinensis* UQM 2034^T^ in a 16S rRNA based tree. The sequences of the six copies of the 16S rRNA gene in the genome differ by up to five nucleotides, and differ by up to 21 nucleotides (1.4%) from the previously published sequence generated from ACM 2034 (AF78775). Most likely this discrepancy is caused by sequencing errors in the publicly available *C. pinensis* 16S sequence.

**Figure 2:**
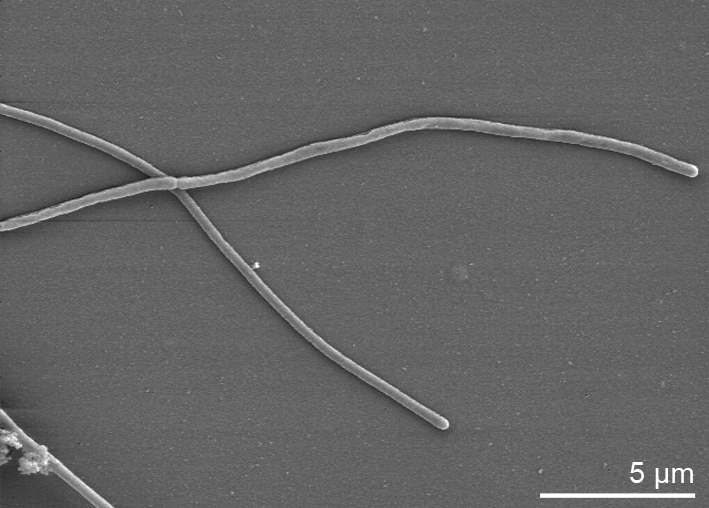
Scanning electron micrograph of *C. pinensis* UQM 2034^T^

Cells of *C. pinensis* stain Gram-negative and form long, filamentous, flexible rods with rounded ends (Table 1). They occur singly and measure 0.5-0.8 by 40 µm in the mature gliding stage. Figure 2 shows cells of *C. pinensis* during cell division before separation. Upon aging, they transform into spherical bodies without the production of fruiting bodies. Myxospores with a diameter of 0.8-0.9 µm are formed. On charcoal yeast extract agar (CYEA) a yellowish pigment is produced. The temperature for growth ranges from 12-37°C with an optimum of 24°C. The optimal pH for growth is 7, but growth is possible in a wide pH range from 4 to 10. NaCl concentrations of 0-1.5% (w/v) are tolerated [1].

**Table 1:** Classification and general features of *C. pinensis* UQM 2034^T^ according to the MIGS recommendations [12]

**MIGS ID**	**Property**	**Term**	**Evidence code**
	Current classification	Domain *Bacteria*	TAS [13]
		Phylum *Bacteroidetes*	TAS [14]
		Class *Sphingobacteria*	TAS [14]
		Order *Sphingobacteriales*	TAS [14]
		Family *‘Chitinophagaceae’*	NAS
		Genus *Chitinophaga*	TAS [1]
		Species *Chitinophaga pinensis*	TAS [1]
		Type strain UQM 2034	TAS [1]
	Gram stain	negative	TAS [1]
	Cell shape	filamentous	TAS [1]
	Motility	gliding	TAS [1]
	Sporulation	myxospores	TAS [1]
	Temperature range	mesophile, 12-40°C	TAS [1]
	Optimum temperature	24°C	TAS [1]
	Salinity	up to 1.5% NaCl	TAS [1]
MIGS-22	Oxygen requirement	aerobic	TAS [1]
	Carbon source	acid production from glucose, lactose and sucrose	TAS [1]
	Energy source	chemoorganotrophic	TAS [1]
MIGS-6	Habitat	soil	TAS [1]
MIGS-15	Biotic relationship	free living	NAS
MIGS-14	Pathogenicity	non pathogenic	NAS
	Biosafety level	1	TAS [15]
	Isolation	pine litter	TAS [1]
MIGS-4	Geographic location	Alderley, Brisbane, Australia	TAS [1]
MIGS-5	Sample collection time	in 1981 or before	NAS
MIGS-4.1 MIGS-4.2	Latitude Longitude	-27.424, 153	TAS
MIGS-4.3	Depth	not reported	
MIGS-4.4	Altitude	not reported	

Evidence codes - IDA: Inferred from Direct Assay (first time in publication); TAS: Traceable Author Statement (i.e., a direct report exists in the literature); NAS: Non-traceable Author Statement (i.e., not directly observed for the living, isolated sample, but based on a generally accepted property for the species, or anecdotal evidence). These evidence codes are from of the Gene Ontology project [16]. If the evidence code is IDA, then the property was observed for a living isolate by one of the authors or an expert mentioned in the acknowledgements.

Strain UQM 2034^T^ produces acid from glucose, lactose, and sucrose. Chitin, casein and gelatin are hydrolyzed, whereas according to Sangkhobol and Skerman (1981) cellulose, starch, alginate, and agar are not hydrolyzed [1]. Nitrate is not reduced to nitrite. *C. pinensis* UQM 2034^T^ produces urease and is catalase and oxidase positive [1,3]. UQM 2034^T^ is susceptible to tetracycline, streptomycin, and chloramphenicol and resistant to neomycin, kanamycin, penicillin G, and erythromycin [1]. *C. pinensis* UQM 2034^T^ is able to lyse *Staphylococcus aureus* cells but not cells of *Escherichia coli*, *Pseudomonas aeruginosa* and *Bacillus subtilis* [1].

### Chemotaxonomy

The fatty acid profile of strain UQM 2034^T^ revealed C_15:0 iso_ (30.4%) and C_16:1ω5c_ (33.2%) as the major fatty acids and C_17:0 iso-3-OH_ (11.5%) and C_15:0 iso-3-OH_ (3.1%) as the major hydroxyl fatty acids. MK-7 is the predominant menaquinone [3]. The polar lipid composition has not been analyzed, but phosphatidylethanolamine is reported for *C. rupis* [7].

## Genome sequencing and annotation

### Genome project history

This organism was selected for sequencing on the basis of its phylogenetic position, and is part of the *** G****enomic* *** E****ncyclopedia of* *** B****acteria and* *** A****rchaea * project [17]. The genome project is deposited in the Genome OnLine Database [11] and the complete genome sequence is deposited in GenBank. Sequencing, finishing and annotation were performed by the DOE Joint Genome Institute (JGI). A summary of the project information is shown in Table 2.

**Table 2:** Genome sequencing project information

**MIGS ID**	**Property**	**Term**
MIGS-31	Finishing quality	Finished
MIGS-28	Libraries used	Two Sanger libraries: 8kb pMCL200 and fosmid pcc1 Fos. One 454 pyrosequence standard library.
MIGS-29	Sequencing platforms	ABI3730, 454 GS FLX
MIGS-31.2	Sequencing coverage	8.9× Sanger; 17.4× pyrosequence
MIGS-30	Assemblers	Newbler, phrap
MIGS-32	Gene calling method	Prodigal, GenePRIMP
	INSDC ID	CP001699
	Genbank Date of Release	August 08, 2009
	GOLD ID	Gc01083
	NCBI project ID	27951
	Database: IMG-GEBA	2501651204
MIGS-13	Source material identifier	DSM 2588
	Project relevance	Tree of Life, GEBA

### Growth conditions and DNA isolation

*C. pinensis* UQM 2034^T^, DSM 2588, was grown in DSMZ medium 67 (CY-Medium) [25] at 22°C. DNA was isolated from 0.5-1 g of cell paste using Qiagen Genomic 500 DNA Kit (Qiagen, Hilden, Germany), with a modified protocol for cell lysis (st/LALMP), as described in Wu *et al.* [17].

### Genome sequencing and assembly

The genome was sequenced using a combination of Sanger and 454 sequencing platforms. All general aspects of library construction and sequencing performed at the JGI can be found at the JGI website (http://www.jgi.doe.gov/). 454 Pyrosequencing reads were assembled using the Newbler assembler version 1.1.02.15 (Roche). Large Newbler contigs were broken into 2,046 overlapping fragments of 1,000 bp and 9,925 of them entered into the final assembly as pseudo-reads. The sequences were assigned quality scores based on Newbler consensus q-scores with modifications to account for overlap redundancy and to adjust inflated q-scores. A hybrid 454/Sanger assembly was made using the parallel phrap assembler (High Performance Software, LLC). Possible mis-assemblies were corrected with Dupfinisher or transposon bombing of bridging clones [18]. Gaps between contigs were closed by editing in Consed, custom primer walk or PCR amplification. A total of 882 Sanger finishing reads were produced to close gaps, to resolve repetitive regions, and to raise the quality of the finished sequence. The error rate of the completed genome sequence is 0.01 in 100,000 nucleotides. Together all sequence types provided 26.3× coverage of the genome. The final assembly contains 91,161 Sanger and 876,658 pyrosequencing reads.

### Genome annotation

Genes were identified using Prodigal [19] as part of the Oak Ridge National Laboratory genome annotation pipeline, followed by a round of manual curation using the JGI GenePRIMP pipeline (http://geneprimp.jgi-psf.org/) [20]. The predicted CDSs were translated and used to search the National Center for Biotechnology Information (NCBI) nonredundant database, UniProt, TIGRFam, Pfam, PRIAM, KEGG, COG, and InterPro databases. Additional gene prediction analysis and functional annotation was performed within the Integrated Microbial Genomes - Expert Review (http://img.jgi.doe.gov/er) platform [21].

## Genome properties

**Figure 3:**
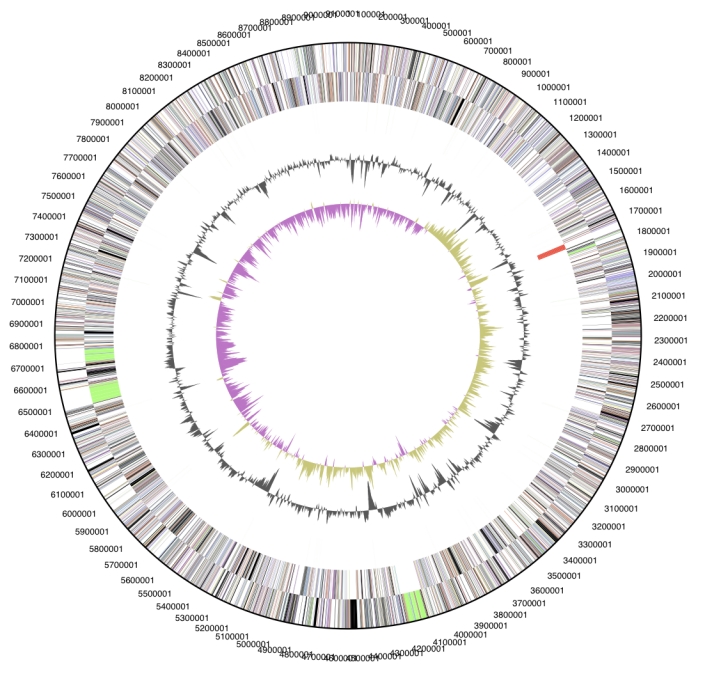
Graphical circular map of the genome. From outside to the center: Genes on forward strand (color by COG categories), Genes on reverse strand (color by COG categories), RNA genes (tRNAs green, rRNAs red, other RNAs black), GC content, GC skew.

The genome is 9,127,347 bp long and comprises one main circular chromosome with a 45.2% GC content (Table 3 and Figure 3). Of the 7,397 genes predicted, 7,302 were protein coding genes, and 95 RNAs. In addition, 110 pseudogenes were also identified. The majority of the protein-coding genes (62.4%) were assigned with a putative function while those remaining were annotated as hypothetical proteins. The distribution of genes into COGs functional categories is presented in Table 4.

**Table 3:** Genome Statistics

**Attribute**	**Value**	**% of Total**
Genome size (bp)	9,127,347	100.00%
DNA Coding region (bp)	8,163,680	89.44%
DNA G+C content (bp)	4,128,036	45.23%
Number of replicons	1	
Extrachromosomal elements	0	
Total genes	7,397	100.00%
RNA genes	95	1.28%
rRNA operons	6	
Protein-coding genes	7,302	98.72%
Pseudo genes	110	1.49%
Genes with function prediction	4,616	62.40%
Genes in paralog clusters	1,705	23.05%
Genes assigned to COGs	4,451	60.17%
Genes assigned Pfam domains	4,685	63.34%
Genes with signal peptides	2,251	30.43%
Genes with transmembrane helices	1,635	22.10%
CRISPR repeats	0	

**Table 4:** Number of genes associated with the general COG functional categories

**Code**	**value**	**%age**	**Description**
J	177	2.4	Translation, ribosomal structure and biogenesis
A	0	0.0	RNA processing and modification
K	613	8.4	Transcription
L	192	2.6	Replication, recombination and repair
B	2	0.0	Chromatin structure and dynamics
D	24	0.3	Cell cycle control, mitosis and meiosis
Y	0	0.0	Nuclear structure
V	123	1.7	Defense mechanisms
T	448	6.1	Signal transduction mechanisms
M	337	4.6	Cell wall/membrane biogenesis
N	25	0.3	Cell motility
Z	0	0.0	Cytoskeleton
W	0	0.0	Extracellular structures
U	64	0.9	Intracellular trafficking and secretion
O	170	2.3	Posttranslational modification, protein turnover, chaperones
C	244	3.3	Energy production and conversion
G	330	4.5	Carbohydrate transport and metabolism
E	301	4.1	Amino acid transport and metabolism
F	91	1.2	Nucleotide transport and metabolism
H	210	2.9	Coenzyme transport and metabolism
I	197	2.7	Lipid transport and metabolism
P	328	4.5	Inorganic ion transport and metabolism
Q	146	2.0	Secondary metabolites biosynthesis, transport and catabolism
R	661	9.1	General function prediction only
S	352	4.8	Function unknown
-	2841	38.9	Not in COGs

## Insights from genome sequence

The predominant characteristic feature of *C. pinensis* is the ability to degrade chitin, a β-1,4-glycosidic linked homopolymer of N-acetyl-D-glucosamine and one of the most abundant polysaccharides in nature. It is a component of fungal cell walls and of arthropod exoskeletons. Chitin is degraded by chitinases (EC 3.2.1.14); endochitinases randomly cleave within the chitin molecule and exochitinases hydrolyze diacetylchitobiose from the end of a chitin chain. Diacetylchitobiose is further degraded to N-acetylglucosamine by the action of N-acetylglucosaminidases (EC 3.2.1.52).

These glycosidic bond hydrolyzing enzymes were grouped in glycoside hydrolase (GH) families based on amino acid sequence similarities (http://www.cazy.org) [22]. For the *C. pinensis* genome 169 glycoside hydrolases belonging to 49 different GH families are predicted; 18 of the predicted glycoside hydrolases belong to GH family 43 which contains xylosidases, xylanases, arabinanases, arabinofuranosidases and galactosidases. 

Because of the chitin degrading ability of *C. pinensis* a great number of chitinases was expected to be encoded in the genome. According to the CAZY-database, exochitinases and endochitinases belong to GH families 18, 19 and 48. As estimated, there were several glycoside hydrolases predicted, which may be involved in chitin degradation*;* five members of GH family 18 (Cpin_2184, Cpin_2186, Cpin_2580, Cpin_3805, Cpin_3919) and three members of GH family 19 (Cpin_5850, Cpin_5553, Cpin_5898). The comparison of the amino acid sequence from these chitinase candidates to the databank BlastP indicated no homologs according to the whole length of the proteins. However, similarities to the known GH family domains were observed.

The search for N-acetylglucosaminidases (EC 3.2.1.52) in the genome of *C. pinensis* revealed gene Cpin_3944 which encodes a protein with a GH family 20 domain. The predicted GH family 20 domain resembles the well characterized catalytic domain of a N-acetylglucosaminidase from *Serratia marcescens* [23]. Further N-acetylhexoaminidases of *C. pinensis* are encoded by the genes Cpin_1798, Cpin_4994 and Cpin_1915.

A second way to degrade chitin was described by Davis and Eveleigh in 1984 [24]. First, the chitin molecule is deacetyliated by deacetylases (EC 3.5.1.41), afterwards chitobiose is released from chitosan by the action of chitosanases (EC 3.2.1.132), finally chitobiose is hydrolyzed by glucosaminidases (EC 3.2.1.52) and glucosamine molecules are released.

One putative chitin deacetylase is encoded in the genome of *C. pinensis*. The deduced amino acid sequence of Cpin_6813 shows a GH family 19 domain and a C-terminal deacetylase domain. Chitosanases that are responsible for the hydrolysis of chitosan are mainly found in GH family 46 but also occur in GH families 5 and 18. In *C. pinensis*, no GH family 46 members were observed, but the presence of nine GHs belonging to family 5 and five members of GH family 18 are predicted. One of these glycoside hydrolases might have a chitosanase function. It remains unclear which pathway *C. pinensis* uses for the degradation of chitin and whether the predicted functions of the proteins match the real functions.

## References

[1] Sangkhobol V Skerman VBD *Chitinophaga*, a new genus of *chitinolytic* myxobacteria. Int J Syst Bacteriol 1981; 31:285-293

[2] Sly LI Taghavi M Fegan M Phylogenetic position of *Chitinophaga pinensis* in the *Flexibacter*-*Bacteroides*-*Cytophaga* phylum. Int J Syst Bacteriol 1999; 49:479-481 10319467 10.1099/00207713-49-2-479

[3] Kämpfer P Young CC Sridhar KR Arun AB Lai WA Shen FT Rekha PD Transfer of [*Flexibacter*] *sancti*, [*Flexibacter*] *filiformis*, [*Flexibacter*] *japonensis* and [*Cytophaga*] *arvensicola* to the genus *Chitinophaga* and description of *Chitinophaga skermanii* sp. nov. Int J Syst Evol Microbiol 2006; 56:2223-2228 10.1099/ijs.0.64359-0 16957125

[4] Lee HG An DS Im WT Liu QM Na JR Cho DH Jin CW Lee ST Yang DC *Chitinophaga ginsengisegetis* sp. nov. and *Chitinophaga ginsengisoli* sp. nov., isolated from soil of a ginseng field in South Korea. Int J Syst Evol Microbiol 2007; 57:1396-1401 10.1099/ijs.0.64688-0 17625164

[5] Kim MK Jung HY *Chitinophaga terrae* sp. nov., isolated from soil. Int J Syst Evol Microbiol 2007; 57:1721-1724 10.1099/ijs.0.64964-0 17684244

[6] Weon HY Yoo SH Kim YJ Son JA Kim BY Kwon SW Koo BS *Chitinophaga niabensis* sp. nov. and *Chitinophaga niastensis* sp. nov., isolated from soil. Int J Syst Evol Microbiol 2009; 59:1267-1271 10.1099/ijs.0.004804-0 19502299

[7] Lee DW Lee JE Lee SD *Chitinophaga rupis* sp. nov., isolated from soil. Int J Syst Evol Microbiol 2009; 59:2830-2833 10.1099/ijs.0.011163-0 19628608

[8] Lee C Grasso C Sharlow MF Multiple sequence alignment using partial order graphs. Bioinformatics 2002; 18:452-464 10.1093/bioinformatics/18.3.452 11934745

[9] Castresana J Selection of conserved blocks from multiple alignments for their use in phylogenetic analysis. Mol Biol Evol 2000; 17:540-552 10742046 10.1093/oxfordjournals.molbev.a026334

[10] Stamatakis A Hoover P Rougemont J A Rapid Bootstrap Algorithm for the RAxML Web Servers. Syst Biol 2008; 57:758-771 10.1080/10635150802429642 18853362

[11] Liolios K Chen IM Mavromatis K Tavernarakis N Hugenholtz P Markowitz VM Kyrpides NC The Genomes On Line Database (GOLD) in 2009: status of genomic and metagenomic projects and their associated metadata. Nucleic Acids Res 2010; 38:D346-D354 10.1093/nar/gkp848 19914934 PMC2808860

[12] Field D Garrity G Gray T Morrison N Selengut J Sterk P Tatusova T Thomson N Allen MJ Angiuoli SV The minimum information about a genome sequence (MIGS) specification. Nat Biotechnol 2008; 26:541-547 10.1038/nbt1360 18464787 PMC2409278

[13] Woese CR Kandler O Wheelis ML Towards a natural system of organisms. Proposal for the domains *Archaea* and *Bacteria*. Proc Natl Acad Sci USA 1990; 87:4576-4579 10.1073/pnas.87.12.4576 2112744 PMC54159

[14] Garrity GM, Holt JG. Taxonomic Outline of the *Archaea* and *Bacteria* In: Garrity GM, Boone DR, Castenholz RW (eds), *Bergey's Manual of Systematic Bacteriology*, Second Edition, Volume 1, Springer, New York, 2001, p. 155-166

[15] Biological Agents. Technical rules for biological agents www.baua.de TRBA 466.

[16] Ashburner M Ball CA Blake JA Botstein D Butler H Cherry JM Davis AP Dolinski K Dwight SS Eppig JT Gene ontology: tool for the unification of biology. The Gene Ontology Consortium. Nat Genet 2000; 25:25-29 10.1038/75556 10802651 PMC3037419

[17] Wu D Hugenholtz P Mavrommatis K Pukall R Dalin E Ivanova N Kunin V Goodwin L Wu M Tindall BJ A phylogeny-driven genomic encyclopedia of *Bacteria* and *Archaea*. Nature 2009; 462:1056-1060 10.1038/nature08656 20033048 PMC3073058

[18] Sims D Brettin T Detter JC Han C Lapidus A Copeland A Glavina Del Rio T Nolan M Chen F Lucas S Complete genome of *Kytococcus sedentarius* type strain (541^T^). Stand Genomic Sci 2009; 1:12-20 10.4056/sigs.761 PMC3035214 21304632

[19] Hyatt D, Chen G-L, LoCascio PF, Land ML, Larimer FW, Hauser LJ. Prodigal: prokaryotic gene recognition and translation initiation site identification. *BMC Genomics* *(in press)* 10.1186/1471-2105-11-119 PMC2848648 20211023

[20] Pati A Ivanova N Mikhailova N Ovchinikova G Hooper SD Lykidis A Kyrpides NC GenePRIMP: A Gene Prediction Improvement Pipeline for microbial genomes. *Nature Methods, (in press)* 10.1038/nmeth.1457 20436475

[21] Markowitz VM Mavromatis K Ivanova NN Chen IMA Chu K Kyrpides NC IMG ER: a system for microbial genome annotation expert review and curation. Bioinformatics 2009; 25:2271-2278 10.1093/bioinformatics/btp393 19561336

[22] Cantarel BL Coutinho PM Rancurel C Bernard T Lombard V Henrissat B The Carbohydrate-Active EnZymes database (CAZy): an expert resource for Glycogenomics. Nucleic Acids Res 2009; 37:D233-D238 10.1093/nar/gkn663 18838391 PMC2686590

[23] Prag G Papanikolau Y Tavlas G Vorgias CE Petratos K Oppenheim AB Structures of chitobiase mutants complexed with the substrate di-N-acetyl-D-glucosamine: the catalytic role of the conserved acidic pair, aspartate 539 and glutamate 540. J Mol Biol 2000; 300:611-617 10.1006/jmbi.2000.3906 10884356

[24] Davis B, Eveleigh DE. Chitosanases: occurrence, production and immobilization. In: Zikalis JP (ed.), *Chitin, Chitosan and Related Enzymes* Academic Press 1984;161-179.

[25] List of growth media used at DSMZ: http://www.dsmz.de/microorganisms/ media_list.php

